# Cumulative Effect of Common Genetic Variants Predicts Incident Type 2 Diabetes: A Study of 21,183 Subjects from Three Large Prospective Cohorts

**Published:** 2011-11-16

**Authors:** Jingyun Yang, Jinying Zhao

**Affiliations:** Department of Biostatistics and Epidemiology, University of Oklahoma Health Sciences Center, Oklahoma City, OK 73104

**Keywords:** Type 2 diabetes, Single nucleotide polymorphism, Genotype risk score, Incident diabetes

## Abstract

Recent genome-wide association studies (GWAS) and their meta-analyses have identified multiple genetic loci that are associated with type 2 diabetes (T2D). Except for variants in the TCF7L2 gene which had a modest effect on diabetic risk, most genetic variants identified so far have only a weak association with diabetes. It is possible that the combination of multiple variants may have a larger effect on disease risk and improve risk prediction. In this study, we focus on SNPs that had been robustly replicated in previous GWAS and were also genotyped in a large sample of 21,183 participants from three large prospective cohorts, including Atherosclerosis Risk in Communities (ARIC) Study, Framingham Offspring Study (FOS) and Multi-Ethnic Study of Atherosclerosis (MESA). Among these, we were able to successfully confirm the associations of 12 SNPs with baseline prevalent T2D in these two cohorts. A genotype risk score (GRS) using these12 risk variants was constructed to examine whether GRS predicts incident diabetes. In a combined meta-analysis, subjects in the highest tertile of GRS had a 1.62-fold increased risk of incident T2D (95% CI, 1.08–2.44, P=1.5×10^−14^) compared to those in the lowest tertile of GRS after adjustment for age, sex, race, smoking, body mass index (BMI), lipids (HDL and LDL) and systolic blood pressure. Moreover, GRS significantly improves risk prediction and reclassification in T2D beyond known risk factors.

## Introduction

Type 2 diabetes (T2D) has reached epidemic proportions in almost all racial/ethnic groups. Currently, over 200 million individuals worldwide suffer from T2D and this number is projected to reach 438 million by 2030 [1]. Although lifestyle and environmental risk factors are believed to be significant contributors to the etiology of diabetes, genetic predisposition has been suggested to play a critical role [2,3]. Recent genome-wide association studies (GWAS) have identified multiple genetic variants for T2D [4–7]. Except for variants in the TCF7L2 gene which had a modest effect on diabetic risk (odds ratio 1.40–1.56) [8,9], most genetic variants identified so far have only a weak association with diabetes (odds ratios ranging from 1.1 to 1.3) [10–12]. It is possible that the combination of multiple variants may have a larger effect and potentially improves risk prediction over established risk factors. This study aims to confirm the association of these SNPs with prevalent T2D and evaluate the prognostic value of a combination of these validated SNPs in predicting incident T2D in a large sample comprising 21,183 subjects from three large prospective cohorts, including The Atherosclerosis Risk in Communities (ARIC) Study, the Framingham Offspring Study (FOS) and the Multi-Ethnic Study of Atherosclerosis (MESA).

## Methods

### Study populations

A detailed description of the study design and methods for each of the three cohorts has been previously reported and is only briefly outlined here.

#### ARIC

a prospective cohort study to investigate the etiology of atherosclerosis and cardiovascular risk factors [13]. From 1987 to 1989, a total of 15,792 subjects were recruited from four US communities: Forsyth County, NC; Jackson, MS; Minneapolis, MN; and Washington County, MD. Participants were examined about every three years. A total of 12,771 subjects with complete genotype and phenotype data were included in the current analyses.

#### FOS

The Framingham Heart Study (FHS), initiated in 1948, recruited 5,209 men and women residing in Framingham, MA. In 1971, the Framingham Offspring Study (FOS) was undertaken to expand the original FHS cohort by including 5,124 children and spouses of the FHS cohort. The design and selection criteria of the FHS and FOS cohorts have been detailed elsewhere [14]. A total of 2,760 participants with complete genotype and phenotype data were included in the present investigation.

#### MESA

a prospective cohort of multi-ethnic groups including 6,814 men and women free of overt cardiovascular disease (CVD) at enrollment. In July 2000, participants were recruited from six field centers: Baltimore, MD; Chicago, IL; Forsyth County, NC; Los Angeles County, CA; New York, NY; and St. Paul, MN. Participants have untaken four clinical examinations through 2007. A total of 5,652 subjects with complete genotype and phenotype data were included in the current analyses.

For all these three cohorts, medical history, physical examination, laboratory tests, and risk factor assessments were performed routinely at each visit [13–15]. Annual follow-up was conducted to collect information on vital status. All participants provided written informed consents, and the institutional review board of participating institutions or clinical sites approved the studies.

### Definition of T2D

Diabetes was defined as a fasting blood glucose level of 126 mg/ dL (7.0mmol/L) or higher or receiving insulin or any hyperglycemic treatment. Incident cases of T2D were defined as participants who were free of overt T2D at enrollment but met the diagnostic criteria in at least one of the clinical exams during follow-up. Incident cases were also verified by review of medical records.

### SNPs selection

We obtained authorization to access GWAS datasets of the three cohorts through dbGaP. Using Affymetrix Genome-Wide Human SNP Assay 6.0 (Affymetrix, Inc., Santa Clara, CA), 841,820 SNPs were genotyped in ARIC and 909,622 SNPs were genotyped in MESA. A total number of 500,568 SNPs were genotyped by Affymetrix 500K Arrays (Affymetrix, Inc., Santa Clara, CA) in FOS.

We did an extensive search for T2D-related SNPs from literature published before December 2010. SNPs that met the following criteria were included in the current analysis: (1) robustly replicated in previous GWAS; (2) genotype data available in the three cohorts (ARIC, FOS and MESA); and (3) positively associated with T2D in all three cohorts.

### Statistical analysis

We first conducted logistic regression analysis to test the association of each individual SNP with prevalent T2D at baseline, adjusting for baseline age, sex, race, BMI, current smoking, high- and low- density lipoprotein (HDL and LDL), and systolic blood pressure (SBP). We then constructed two weighted genetic risk scores (GRS) by summing the number of risk alleles (0, 1 or 2) of each SNP that was independently associated with prevalent diabetes, with one weight being the corresponding effect sizes defined by this study, and another being the effect sizes reported in literature [16–20]. Since results obtained by the two weights are similar, we choose to present the results obtained using GRS weighted by the effect sizes reported in literature. Analysis was first done in each cohort separately and results were then combined using a random-effects meta-analysis with inverse variance weight. Heterogeneity across the study cohorts was assessed using Q statistics [21]. Multiple testing was adjusted using Bonferroni correction. We did not test the association of these SNPs in FOS because of the limited number of prevalent diabetics (0.5%) in this cohort.

To determine the prognostic value of the weighted GRS (categorized in tertiles) in predicting incident T2D, we performed multivariate Cox proportional hazards regression, adjusting for covariates listed above. The assumption of proportional hazards was tested using scaled Schoenfeld residuals [22]. For each cohort, we estimated the hazard ratio (HR) and corresponding 95% confidence interval (CI) by comparing the highest to the lowest tertile of GRS, and tested the null hypothesis of no linear trend over the tertiles using Wald test. We conducted random-effects meta-analyses to combine results of the three cohorts. Modified inverse normal method [23] was used to combine p-values from the meta-analyses.

To further evaluate the potential value of GRS in risk prediction, we also examined its association with prevalent diabetes at the end of follow-up (i.e., baseline diabetics plus incident diabetes by the end of follow-up) using logistic regression, controlling for covariates described above. Participants were classified into four categories (<5%, 5–10%, 10–20% and ≥20%) based on predicted probabilities with or without GRS. First, we compared the difference in area under the ROC curve (AUC) using the non-parametric approach based on generalized U-statistics [24]. Second, we calculated the integrated discrimination improvement (IDI) and the net reclassification improvement (NRI), as suggested by Pencina et al. [25]. All continuous variables are log-transformed to increase normality. An association was considered to be significant if adjusted p-value<0.05. Analyses were done using Plink [26], SAS version 9.2 (SAS Institute Inc., Cary, NC, USA), R statistical package (version 2.11.1) or Matlab 7.10.0.499 (The Math Works, Inc., Natick, MA, USA).

## Results

### Baseline characteristics

A total of 21,183 subjects, including 12,771 from ARIC, 2,760 from FOS, and 5,652 from MESA, were included in the current analyses. Baseline characteristics of the study participants are shown in Table 1. On average, participants from FOS are younger, less obese and more likely to be current smokers (all p’s <0.0001). Prevalence T2D at baseline was 11.6% and 12.7% in ARIC and MESA, respectively. Baseline prevalence of T2D in FOS was very low (0.5%), possibly due to younger age of the study participants at enrollment (mean age 33.7 years old). Incident diabetes rates were similar among participants from ARIC and FOS but higher than those from MESA (9.8% in ARIC and FOS vs. 7.1% in MESA). The median of total follow-up time was 16.1 years, 32.3 years and 6.5 years for ARIC, FOS, and MESA, respectively. The mean GRS scores were 13.3±4.2, 11.7±2.8 and 14.2±3.9 for ARIC, FOS and MESA, respectively. Diabetic patients on average had a higher GRS than nondiabetics (15.3 vs. 13.3, P<0.0001 in the combined sample).

### Single SNP association with prevalent diabetes at baseline

We confirmed the associations of 12 SNPs with prevalent diabetes in ARIC and MESA (Table 2), after correction for multiple testing using Bonferroni adjustment. Out of the 12 SNPs, 9 were genotyped in FOS. The remaining 3 SNPs were imputed by the computer program Impute2 (version 2.1.2) using the 1,000 Genomes Project (2010 interim, Dec., 2010) and the HapMap Phase 3 as reference populations [27].

As anticipated, the genotype risk score constructed using these 12 SNPs was significantly associated with an increased risk for diabetes at baseline. After adjustment for risk factors listed above, the odds ratio (OR, highest vs. lowest tertile) for prevalent T2D was 1.78 (95% CI, 1.45–2.19; P<0.0001) in ARIC, and 2.63 (95% CI, 1.97–3.51; P<0.0001) in MESA. We did not test the association of GRS with prevalent T2D in FOS due to limited number of diabetic patients at baseline. Meta-analyses of the two cohorts also revealed a significant association of GRS with prevalent diabetes at enrollment (OR=2.15, 95% CI, 1.47– 3.14; P=1.5 × 10^−10^) (Table 3).

### Association of GRS with incident diabetes

The median follow-up time to the first T2D event was 8.8 years, 26.3 years and 4.7 years for ARIC, FOS, and MESA, respectively. Table 3 shows the association of GRS with incident T2D. Compared with those in the bottom tertile of GRS, subjects in the top tertile had 1.31 times (95% CI 1.08–1.57; P= 0.0008) and 2.64 times (95% CI 1.84–3.79; P<0.0001) increased risk of T2D in ARIC and FOS, respectively. But GRS did not predict incident diabetes in MESA (P=0.26). Meta-analyses combining samples from all three studies identified a 1.62 times increased risk (95% CI, 1.08–2.44; P= 0.02) in comparing subjects in the highest tertile of GRS to those in the lowest tertile. We detected a significant heterogeneity across the three studies (Q=12.04, P=0.003), but this should not be a concern for our analyses because random effects meta-analysis used in this study takes into account both within-and between-study variability [28].

Additionally, we examined whether the observed association between GRS and incident diabetes was driven by rs7901695 in the gene TCF7L2, the strongest diabetic gene reported so far. After removing rs7901695, the hazard ratio (HR) for incident diabetes was only slightly attenuated in each cohort, with HR=1.28 (95% CI, 1.06–1.54; P=0.025) in ARIC, HR=2.24 (95% CI, 1.58–3.16; P<0.0001) in FOS and HR=1.18 (95% CI, 0.85–1.66; P=0.32) in MESA. Thus, the observed association of GRS with incident diabetes is unlikely dominated by this SNP.

**Figure 1:**
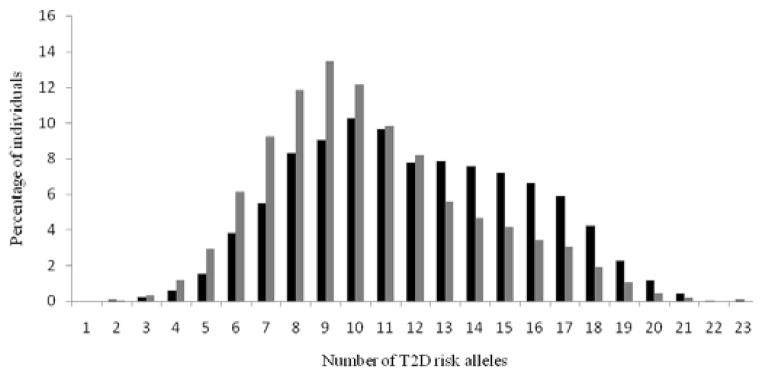
Distribution of risk alleles in diabetic (black bars) and nondiabetic subjects (gray bars).

**Figure 2:**
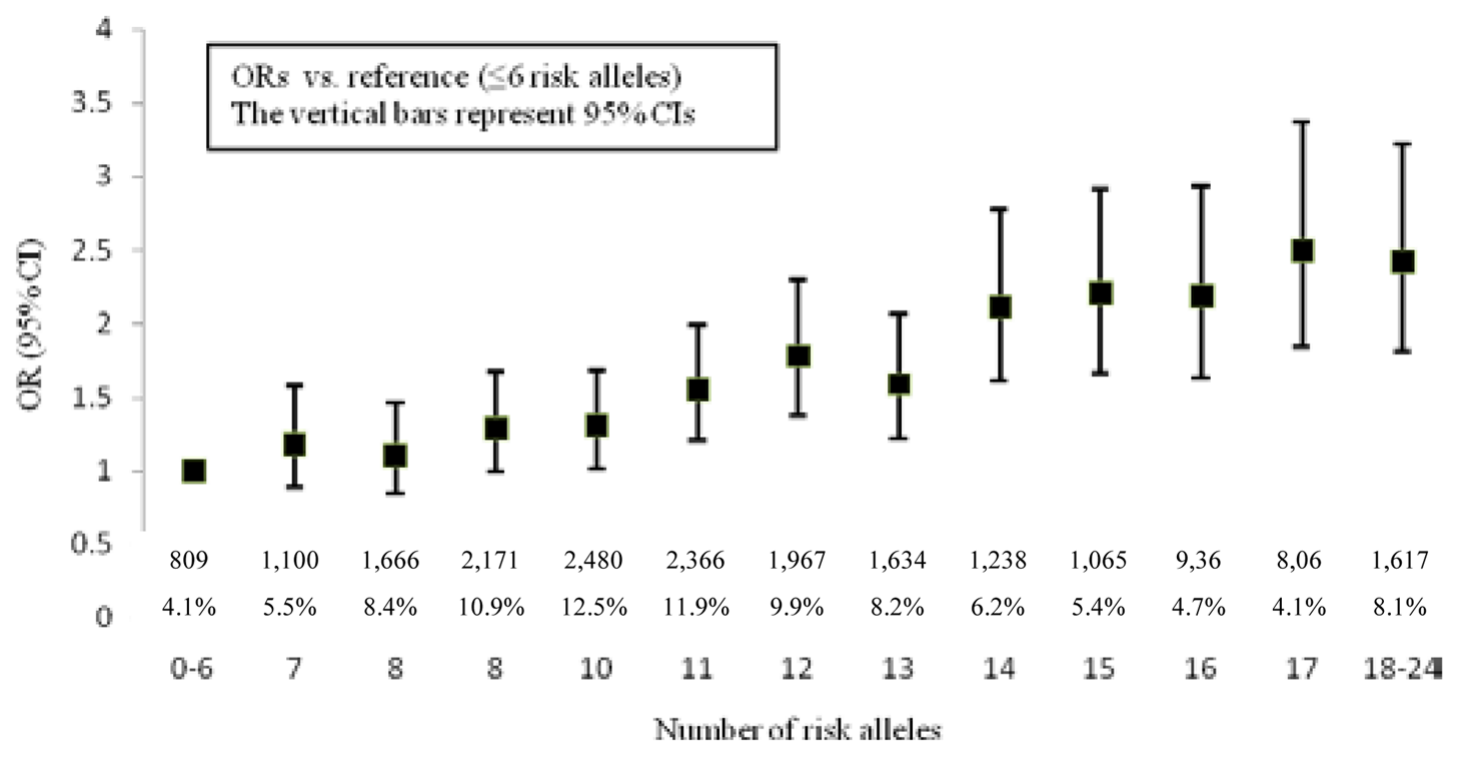
Odds ratios for type 2 diabetes according to the number of risk alleles carried.

**Figure 3:**
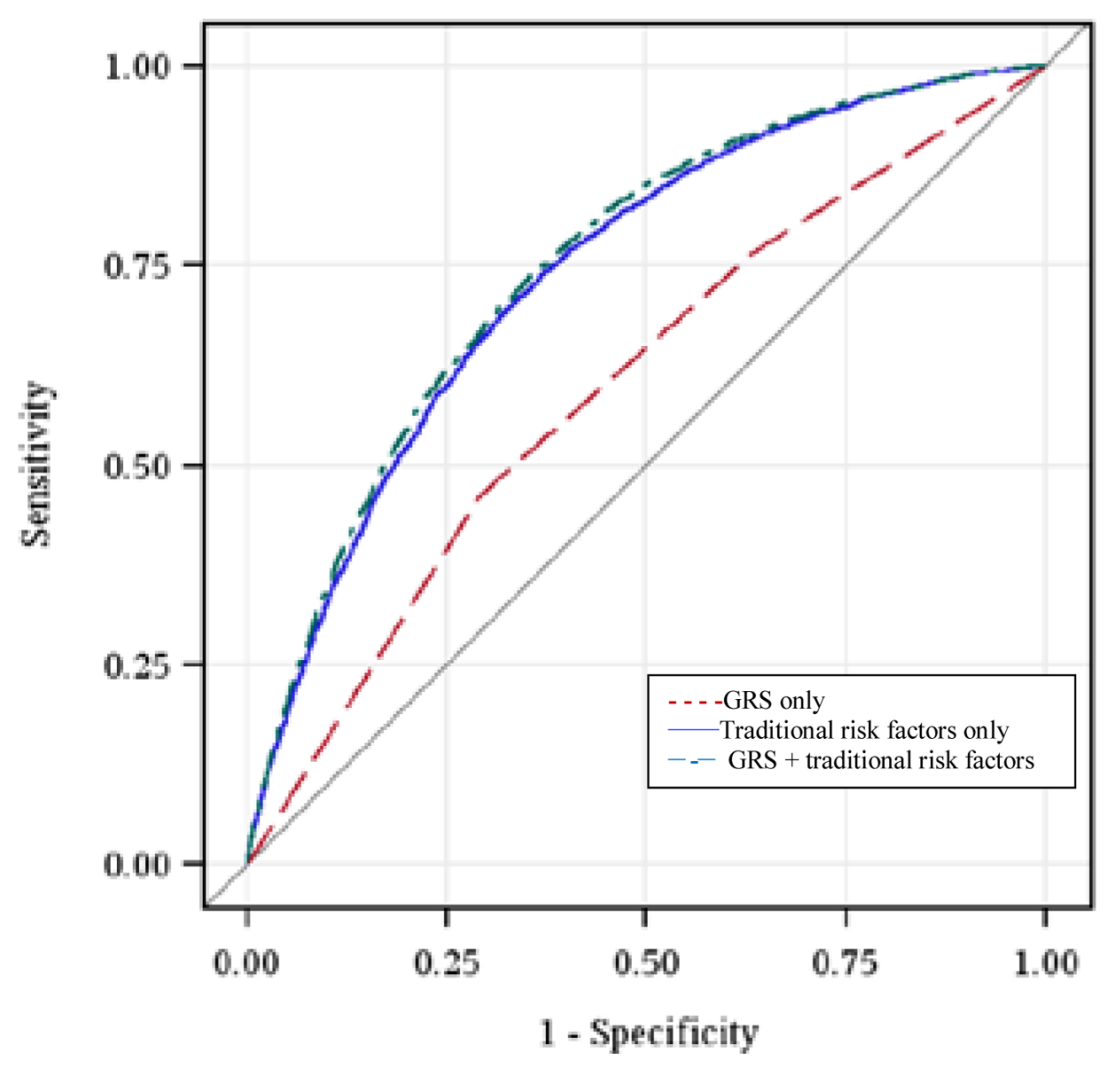
ROC curves for prediction of T2D based on traditional risk factors, GRS and both.

Figure 1 shows the distribution of the number of risk alleles carried among the study participants by diabetes status at the end of study follow-up. The distribution is approximately normal, with diabetics carrying more risk alleles than nondiabetics. Figure 2 shows ORs associated with carrying increased number of risk alleles compared with reference group (0–6 risk alleles). Individuals carrying 18 or more risk alleles were more than twice as likely to have T2D as those carrying 6 or less risk alleles (OR=2.42, 95% CI: 1.82–3.42; P<0.0001). Table 3 shows the significant association of GRS with prevalent T2D at end of follow-up in each cohort as well as the combined sample. The adjusted-OR (highest vs. lowest tertile of GRS) was 1.64 (95% CI: 1.39–1.92; P<0.0001) for ARIC, 2.65 (95% CI: 1.78–3.96; P<0.0001) for FOS, and 2.18 (95% CI: 1.71–2.78; P<0.0001) for MESA. Meta-analysis of the three cohorts showed a two-fold increased risk for diabetes (OR=2.08, 95% CI: 1.59–2.74; P<0.0001) after adjusting for covariates. The AUC estimates with and without GRS, adjusting for known risk factors, was 0.74 and 0.75, respectively (P<0.0001, Figure 3), indicating that including GRS slightly, yet significantly, improves risk prediction for T2D beyond established risk factors.

Results for risk reclassification of prevalent T2D were shown in Tables 4. A total of 276 participants (29%) with T2D at 10–20% risk estimated using only known coronary risk factors were reclassified into ≥20% category when GRS was added to the model, and 932 participants (20%) without T2D were reclassified from ≥20% into the 10–20% category when GRS was included. Both IDI (IDI=0.016; P<0.0001) and the NRI (NRI=0.076; P<0.0001) were highly significant, suggesting that including GRS significantly improves the discriminatory ability for risk prediction of T2D over known risk factors.

**Figure 4:**
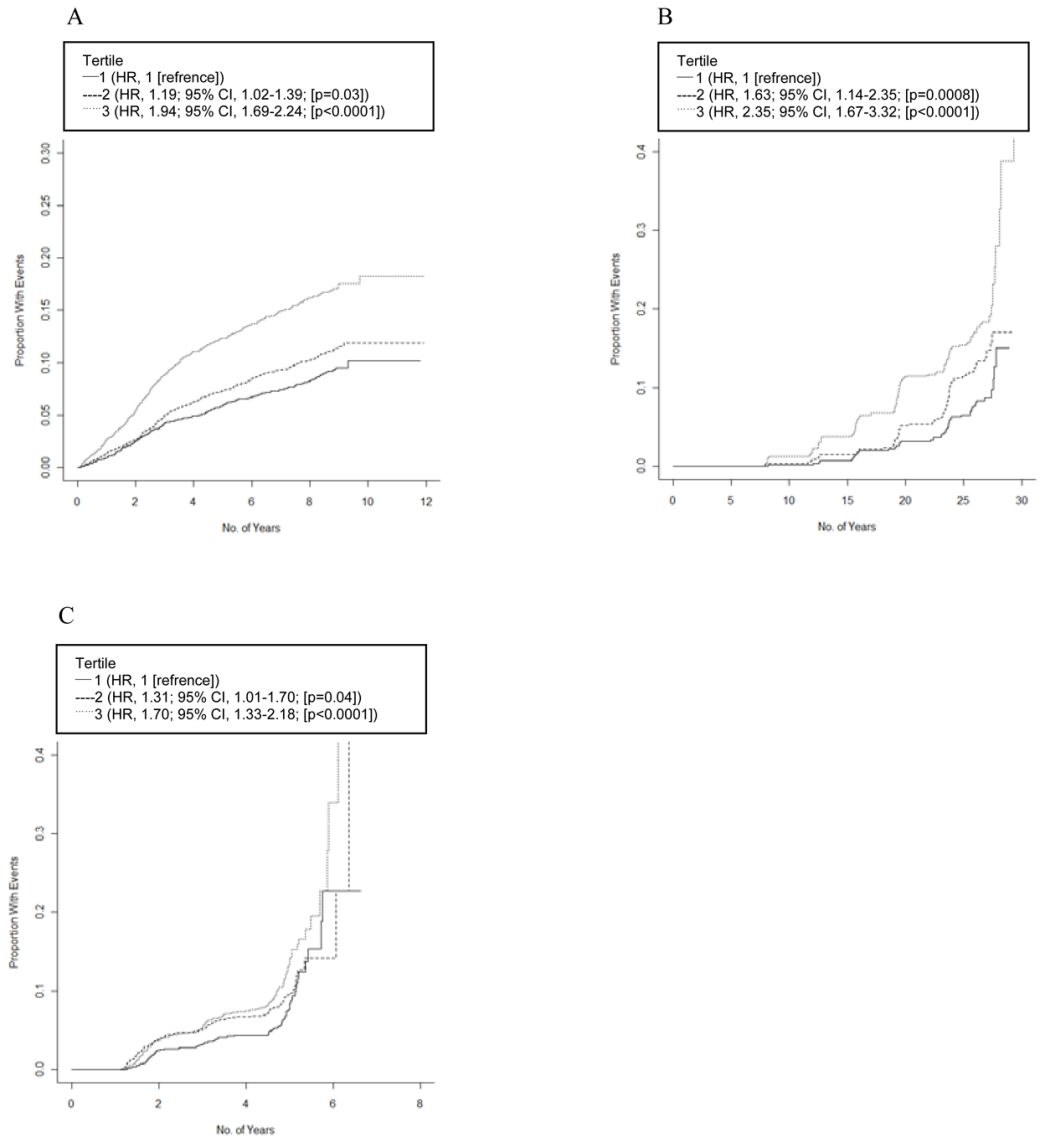
Cumulative incidence of type 2 diabetes by GRS tertiles (A: ARIC; B: FOS; C: MESA).

Figure 4 shows the estimated unadjusted survival curves by GRS tertiles. There was a clear trend toward increasing risk of diabetes with a higher GRS, with the highest tertile of GRS presenting a significant HR of 1.94 (95% CI, 1.69–2.24; P<0.0001) in ARIC, 2.35 (95% CI, 1.67–3.32; P<0.0001) in FOS, and 1.70 (95% CI, 1.33–2.18; P<0.0001) in MESA.

## Discussion

In a sample of 21,183 subjects from three large prospective cohorts, we replicated the individual association of 12 GWAS-identified SNPs with prevalent T2D and demonstrated that cumulative effect of these 12 SNPs significantly predicts incident T2D independent of multiple risk factors. Moreover, the genotype risk score of these 12 SNPs significantly improves risk prediction and reclassification of T2D over known cardiovascular risk factors. To our best knowledge, this is the first investigation of its kind in a large sample of longitudinal cohorts from the U.S communities.

Except for TCF7L2*,* most SNPs identified so far displayed weak or modest effects on diabetes [4]. In line with previous findings, our study also demonstrated that a SNP in TCF7L2 (rs7901695) showed strong association with T2D. Specially, subjects carrying the “C” risk allele of rs7901695 had a 1.42 times increased risk (95% CI, 1.32–1.51; P=1.27 × 10^−24^) for T2D compared to those carrying the “T” allele. In addition, our study elucidated a cumulative effect of multiple diabetes-susceptible loci in relation to diabetic risk. Previous studies showed that including genetic information of risk variants provided only limited value in prediction of T2D beyond classical risk factors [29,30].

Our study, however, demonstrated that knowledge of common genetic variants significantly improves risk prediction and reclassification of T2D beyond clinical risk factors. This finding is corroborated by a recent study indicating that inclusion of common genetic variations appropriately reclassifies younger patients with T2D [31]. Our study has a few limitations. First, we only included a subset of diabetes-associated SNPs available in our study sample. Nonetheless, previous study showed that including more associated SNPs might not contribute much to risk prediction [32]. Second, the three cohorts used in our analyses were undertaken in U.S. communities and hence our results might not be generalized to ethnic groups different from those used in this study. Third, despite the large number of subjects involved, our analyses may still be underpowered to detect variants of small effect and rare variants. Fourth, using GRS to evaluate joint effects of multiple risk variants assumes independence among the studied SNPs. However, genetic interactions (e.g., gene-gene) are known to play important roles in the etiology of complex disorders including diabetes [33,34]. Finally, despite adjustment for race in all statistical analyses, we cannot fully exclude the possibility of population stratification or confounding by ancestry in our samples.

In summary, we confirmed the associations of 12 GWAS-identified diabetic SNPs with prevalent T2D in three well-phenotyped prospective cohorts, and further demonstrated that the combined effects of these diabetic risk variants significantly predict incident diabetes independent of known cardiovascular risk factors. Moreover, a GRS comprising these 12 SNPs also significantly improves risk prediction and reclassification for T2D over classical risk factors. However, because of the unknown pathophysiology of these risk variants, the clinical implications of our findings remain to be determined. Given the high heritability of diabetes, the potential roles of more common variants and other sources of variation, such as rare variants, copy number variation and epigenetics, should also be investigated in future research.

## Figures and Tables

**Table 1:** Demographic information of the study participants.

	ARIC (n=12,771)	FOS (n=2,760)	MESA (n=5,652)	P-value
Sex				
Male (n, %)	5704 (44.7%)	1258 (45.6%)	2753 (48.7%)	<0.0001
Age (mean±SD, in years)	54.1± 5.7	33.7±9.3	62.4±10.3	<0.0001
Ethnicity				<0.0001
White	9633 (75.4%)	2696 (97.7%)	2224 (39.4)	
Black	3138 (24.6%)	7 (0.3%)	1386 (24.5%)	
Other	-	57 (2%)	2042 (36.1%)	
HDL (mg/dl)	51.5±16.9	51.5±14.5	50.8±14.8	0.009
LDL (mg/dl)	137.9±39.2	120.1±32.9	117.2±31.6	<0.0001
SBP (mm Hg)	120.9±18.5	119.0±14.2	134.4±19.0	<0.0001
DBP (mm Hg)	73.6±11.2	77.1±9.9	75.2 ±10.0	<0.0001
BMI (kg/m^2^)	27.7±5.3	24.9±4.1	28.3±5.5	<0.0001
Current smoker (n, %)	3291 (25.8%)	1065 (38.7%)	708 (12.5%)	<0.0001
Follow-up time (in years)				
Mean±SD	15.4±2.9	31.0±4.0	6.1±1.2	<0.0001
Median	16.1	32.3	6.5	<0.0001
Baseline T2D prevalence (n, %)	1484 (11.6%)	13 (0.5%)	717 (12.7%)	<0.0001
T2D incidence (n, %)	1249 (9.8%)	272 (9.9%)	400 (7.1%)	<0.0001
GRS	13.3±4.2	11.7±2.8	14.2±3.9	<0.0001

**Abbreviations:** HDL: High Density Lipoprotein; LDL: Low-Density Lipoprotein; SBP: Systolic Blood Pressure; DBP: Diastolic Blood Pressure; BMI: Body Mass Index; T2D: Type 2 Diabetes; GRS: Genotype Risk Score

**Table 2:** Significant associations of 12 GWAS-identified risk variants with prevalent T2D at enrollment^a^.

SNP	Gene	Risk allele	ARIC		MESA		Pooled^b^	
			OR (95% CI)	P-value	OR (95% CI)	p-value	OR	P-value
rs10923931	*NOTCH2* *ADAM30*	**T**	1.43 (1.30–1.57)	2.1×10^−14^	1.35 (1.16–1.57)	1.32×10^−4^	1.41 (1.30–1.52)	1.54×10^−17^
rs780094	*GCKR* *TMEM195*	**C**	1.33 (1.22–1.44)	2.5×10^−11^	1.30 (1.14–1.47)	5.40×10^−5^	1.32 (1.23–1.41)	6.63×10^−15^
rs1801282	*PPARG*	**C**	1.53 (1.32–1.77)	1.48×10^−8^	1.53 (1.19–1.96)	9.66×10^−4^	1.53 (1.35–1.74)	5.56×10^−11^
rs1470579	*IGF2BP2*	**G**	1.50 (1.39–1.61)	3.27×10^−28^	1.24 (1.11–1.38)	1.95×10^−4^	1.37 (1.13–1.65)	1.2×10^−3^
rs10946398	*CDKAL1*	**G**	1.34 (1.24–1.44)	7.43×10^−14^	1.44 (1.28–1.62)	1.03×10^−9^	1.37 (1.28–1.47)	3.28×10^−18^
rs7756992	*CDKAL1*	**C**	1.42 (1.32–1.53)	7.98×10^−20^	1.41 (1.25–1.58)	9.14×10^−9^	1.42 (1.33–1.51)	4.98×10^−27^
rs864745	*JAZF1*	**T**	1.31 (1.22–1.42)	5.56×10^−12^	1.29 (1.14–1.46)	4.19×10^−5^	1.31 (1.22–1.40)	1.07×10^−15^
rs564398	*CDKN2B*	**A**	1.46 (1.34–1.59)	4.37×10^−19^	1.38 (1.20–1.59)	9.3×10^−6^	1.44 (1.34–1.55)	2.54×10^−23^
rs7901695	*TCF7L2*	**C**	1.45 (1.34–1.57)	1.44×10^−20^	1.34 (1.19–1.52)	1.36×10^−6^	1.42 (1.32–1.51)	1.27×10^−24^
rs1495377	*NR*	**G**	1.25 (1.16–1.35)	1.27×10^−08^	1.37 (1.21–1.56)	5.23×10^−7^	1.29 (1.18–1.41)	1.04×10^−8^
rs11634397	*ZFAND6*	**T**	1.32 (1.22–1.42)	1.44×10^−12^	1.23 (1.10–1.38)	2.17×10^−4^	1.29 (1.21–1.37)	2.23×10^−15^
rs8042680	*PRC1*	**A**	1.47 (1.37–1.58)	8.03×10^−27^	1.56 (1.39–1.75)	1.21×10^−13^	1.49 (1.41–1.59)	9.82×10^−39^

a Multiple testing was adjusted using Bonferroni correction

b Results from MESA and ARIC were combined using random-effects meta-analysis

**Table 3:** Association between genotype risk score, T2D prevalence and incident T2D.

Association of GRS with T2D prevalence at enrollment		
Study	OR (95% CI, tertile 3 vs. tertile 1; P-value)	P for trend
ARIC	1.78 (1.45–2.19; P<0.0001)	<0.0001
FOS	--	--
MESA	2.63 (1.97–3.51; P<0.0001)	<0.0001
Pooled	2.15 (1.47–3.14; P<0.0001)	1.5×10^−10^
Association of GRS with T2D prevalence at the end of follow-up		
Study	OR (95% CI, tertile 3 vs. tertile 1; P-value)	P for trend
ARIC	1.64 (1.39–1.92; P<0.0001)	<0.0001
FOS	2.65 (1.78–3.96; P<0.0001)	<0.0001
MESA	2.18 (1.71–2.78; P<0.0001)	<0.0001
Pooled	2.08 (1.59–2.74; P<0.0001)	4.9×10^−12^
Association of GRS with incident T2D		
Study	HR (95% CI, tertile 3 vs. tertile 1; P-value)	P for trend
ARIC	1.31 (1.08–1.57; P=0.0008)	0.0008
FOS	2.64 (1.84–3.79; P<0.0001)	<0.0001
MESA	1.32 (0.95–1.83; P=0.10)	0.26
Pooled	1.62 (1.08–2.44; P=0.02)	1.5×10^−14^

All ORs and P-values adjusted for age, sex, race, current smoking, BMI, HDL, LDL and SBP

**Table 4:** Reclassification of individuals on predicted risk of T2D with and without GRS (pooled).

Predicted risk^a^	Predicted risk based on known risk factors + GRS			
	0–5%	5–10%	10–20%	≥20%
Cases				
0–5%	17 (57%)	13 (43%)	0	0
5–10%	18 (10%)	107 (61%)	50 (29%)	0
10–20%	0	91 (10%)	582 (61%)	276 (29%)
≥ 20%	0	0	219 (9%)	2269 (91%)
Non-cases				
0–5%	557 (84%)	109 (16%)	0	0
5–10%	497 (17%)	1816 (64%)	533 (19%)	
10–20%	0	1114 (19%)	3796 (66%)	879 (15%)
≥ 20%	0	0	932 (20%)	3738 (80%)
Net reclassification improvement of GRS for T2D				
	Individual reclassified		NRI	P-value
	Up	Down		
Cases	339	328	0.003	0.67
Non-cases	1518	2543	0.073	<0.0001
Total	-	-	0.076	<0.0001

a Predicted risk based on known risk factors only, including age, sex, race, current smoking, BMI, HDL, LDL and SBP

## References

[1] Wild S, Roglic G, Green A, Sicree R, King H (2004). Global prevalence of diabetes: estimates for the year 2000 and projections for 2030. Diabetes Care.

[2] Barroso I (2005). Genetics of Type 2 diabetes. Diabet Med.

[3] Herder C, Roden M (2011). Genetics of type 2 diabetes: pathophysiologic and clinical relevance. Eur J Clin Invest.

[4] McCarthy MI, Zeggini E (2009). Genome-wide association studies in type 2 diabetes. Curr Diab Rep.

[5] Zeggini E, Scott LJ, Saxena R, Voight BF, Marchini JL (2008). Meta-analysis of genome-wide association data and large-scale replication identifies additional susceptibility loci for type 2 diabetes. Nat Genet.

[6] Vimaleswaran KS, Loos RJ (2010). Progress in the genetics of common obesity and type 2 diabetes. Expert Rev Mol Med.

[7] McCarthy MI (2008). Casting a wider net for diabetes susceptibility genes. Nat Genet.

[8] Saxena R, Gianniny L, Burtt NP, Lyssenko V, Giuducci C (2006). Common single nucleotide polymorphisms in TCF7L2 are reproducibly associated with type 2 diabetes and reduce the insulin response to glucose in nondiabetic individuals. Diabetes.

[9] Grant SF, Thorleifsson G, Reynisdottir I, Benediktsson R, Manolescu A (2006). Variant of transcription factor 7-like 2 (TCF7L2) gene confers risk of type 2 diabetes. Nat Genet.

[10] Barroso I, Luan J, Sandhu MS, Franks PW, Crowley V (2006). Meta-analysis of the Gly482Ser variant in PPARGC1A in type 2 diabetes and related phenotypes. Diabetologia.

[11] Weedon MN, Owen KR, Shields B, Hitman G, Walker M (2005). A large-scale association analysis of common variation of the HNF1alpha gene with type 2 diabetes in the U.K. Caucasian population. Diabetes.

[12] Cauchi S, El Achhab Y, Choquet H, Dina C, Krempler F (2007). TCF7L2 is reproducibly associated with type 2 diabetes in various ethnic groups: a global meta-analysis. J Mol Med.

[13] The ARIC investigators (1989). The Atherosclerosis Risk in Communities (ARIC) Study: design and objectives. Am J Epidemiol.

[14] Kannel WB, Feinleib M, McNamara PM, Garrison RJ, Castelli WP (1979). An investigation of coronary heart disease in families. The Framingham offspring study. Am J Epidemiol.

[15] Bild DE, Bluemke DA, Burke GL, Detrano R, Diez Roux AV (2002). Multiethnic study of atherosclerosis: objectives and design. Am J Epidemiol.

[16] Voight BF, Scott LJ, Steinthorsdottir V, Morris AP, Dina C (2010). Twelve type 2 diabetes susceptibility loci identified through large-scale association analysis. Nat Genet.

[17] Dupuis J, Langenberg C, Prokopenko I, Saxena R, Soranzo N (2010). New genetic loci implicated in fasting glucose homeostasis and their impact on type 2 diabetes risk. Nat Genet.

[18] Scott LJ, Mohlke KL, Bonnycastle LL, Willer CJ, Li Y (2007). A genome-wide association study of type 2 diabetes in Finns detects multiple susceptibility variants. Science.

[19] Shu XO, Long J, Cai Q, Qi L, Xiang YB (2010). Identification of new genetic risk variants for type 2 diabetes. PLoS Genet.

[20] Horikawa Y, Miyake K, Yasuda K, Enya M, Hirota Y (2008). Replication of genome-wide association studies of type 2 diabetes susceptibility in Japan. J Clin Endocrinol Metab.

[21] Cochran WG (1954). The Combination of Estimates from Different Experiments. Biometrics.

[22] Schoenfeld D (1982). Partial Residuals for the Proportional Hazards Regression-Model. Biometrika.

[23] Hartung J (1999). A note on combining dependent tests of significance. Biometrical Journal.

[24] DeLong ER, DeLong DM, Clarke-Pearson DL (1988). Comparing the areas under two or more correlated receiver operating characteristic curves: a nonparametric approach. Biometrics.

[25] Pencina MJ, D’Agostino RB, D’Agostino RB, Vasan RS (2008). Evaluating the added predictive ability of a new marker: from area under the ROC curve to reclassification and beyond. Stat Med.

[26] Purcell S, Neale B, Todd-Brown K, Thomas L, Ferreira MA (2007). PLINK: a tool set for whole-genome association and population-based linkage analyses. Am J Hum Genet.

[27] Altshuler DM, Gibbs RA, Peltonen L, Dermitzakis E, Schaffner SF (2010). Integrating common and rare genetic variation in diverse human populations. Nature.

[28] Hedges LV, Vevea JL (1998). Fixed- and random-effects models in meta-analysis. Psychological Methods.

[29] Lango H, Palmer CN, Morris AD, Zeggini E, Hattersley AT (2008). Assessing the combined impact of 18 common genetic variants of modest effect sizes on type 2 diabetes risk. Diabetes.

[30] van Hoek M, Dehghan A, Witteman JC, van Duijn CM, Uitterlinden AG (2008). Predicting type 2 diabetes based on polymorphisms from genome-wide association studies: a population-based study. Diabetes.

[31] de Miguel-Yanes JM, Shrader P, Pencina MJ, Fox CS, Manning AK (2011). Genetic risk reclassification for type 2 diabetes by age below or above 50 years using 40 type 2 diabetes risk single nucleotide polymorphisms. Diabetes Care.

[32] Paynter NP, Chasman DI, Pare G, Buring JE, Cook NR (2010). Association between a literature-based genetic risk score and cardiovascular events in women. JAMA.

[33] Moore JH (2003). The ubiquitous nature of epistasis in determining susceptibility to common human diseases. Hum Hered.

[34] Hunter DJ (2005). Gene-environment interactions in human diseases. Nat Rev Genet.

